# Successful Medical Therapy for Hypophosphatemic Rickets due to Mitochondrial Complex I Deficiency Induced de Toni-Debré-Fanconi Syndrome

**DOI:** 10.1155/2013/354314

**Published:** 2013-12-10

**Authors:** Sasigarn A. Bowden, Hiren P. Patel, Allan Beebe, Kim L. McBride

**Affiliations:** ^1^Division of Endocrinology, Nationwide Children's Hospital, The Ohio State University College of Medicine, 700 Children's Drive, Columbus, OH 43205, USA; ^2^Division of Nephrology, Nationwide Children's Hospital, The Ohio State University College of Medicine, Columbus, OH 43205, USA; ^3^Division of Orthopedics, Nationwide Children's Hospital, The Ohio State University College of Medicine, Columbus, OH 43205, USA; ^4^Center for Cardiovascular and Pulmonary Research, Department of Pediatrics, Nationwide Children's Hospital, The Ohio State University College of Medicine, Columbus, OH 43205, USA

## Abstract

Primary de Toni-Debré-Fanconi syndrome is a non-FGF23-mediated hypophosphatemic disorder due to a primary defect in renal proximal tubule cell function resulting in hyperphosphaturia, renal tubular acidosis, glycosuria, and generalized aminoaciduria. The orthopaedic sequela and response to treatment of this rare disorder are limited in the literature. Herein we report a long term followup of a 10-year-old female presenting at 1 year of age with rickets initially misdiagnosed as vitamin D deficiency rickets. She was referred to the metabolic bone and genetics clinics at 5 years of age with severe genu valgum deformities of 24 degrees and worsening rickets. She had polyuria, polydipsia, enuresis, and bone pain. Diagnosis of hypophosphatemic rickets due to de Toni-Debré-Fanconi syndrome was subsequently made. Respiratory chain enzyme analysis identified a complex I mitochondrial deficiency as the underlying cause. She was treated with phosphate (50–70 mg/kg/day), calcitriol (30 ng/kg/day), and sodium citrate with resolution of bone pain and normal growth. By 10 years of age, her genu valgus deformities were 4 degrees with healing of rickets. Her excellent orthopaedic outcome despite late proper medical therapy is likely due to the intrinsic renal tubular defect that is more responsive to combined alkali, phosphate, and calcitriol therapy.

## 1. Introduction

Hypophosphatemic rickets (HR) is a group of diseases sharing similar biochemical phenotypes including excessive renal phosphate wasting, low serum phosphate, and inappropriately low or normal serum 1,25-dihydroxyvitamin D (1,25-OHD) for the given level of hypophosphatemia. After the discovery of fibroblast growth factor 23 (FGF-23), a phosphaturic factor, that is produced by osteocytes and osteoblasts and regulates phosphate homeostasis, HR is now divided into 2 groups: FGF23-mediated and non-FGF23-mediated [1]. FGF23-mediated HR consists of a number of inherited disorders such as X-linked hypophosphatemic rickets (XLH), the most common form, or less common forms such as autosomal dominant/autosomal recessive hypophosphatemic rickets. Aberrant production of FGF-23 is seen in tumor-induced osteomalacia or fibrous dysplasia. The non-FGF23-mediated hypophosphatemic rickets includes hypophosphatemic rickets with hypercalciuria, and renal Fanconi syndrome.

Primary de Toni-Debré-Fanconi syndrome is due to a generalized defect in renal proximal tubule cell function and is characterized by hypophosphatemic rickets, renal tubular acidosis, renal glycosuria, and generalized aminoaciduria. The orthopaedic sequela of this rare disorder in the literature is scarce. Most treatment outcomes of hypophosphatemic rickets were described in familial form, particularly in X-linked hypophosphatemic rickets (XLH). We present an interesting case of a 10-year-old female with primary de Toni-Debré-Fanconi syndrome presenting with significant genu valgum as a result of hypophosphatemic rickets, for which the treatment was delayed, but yet had a satisfactory growth and orthopaedic outcome after treatment with phosphate, calcitriol, and sodium citrate. The underlying mitochondrial disorder as a cause of Fanconi syndrome is also discussed.

## 2. Case Presentation

**Figure 1:**
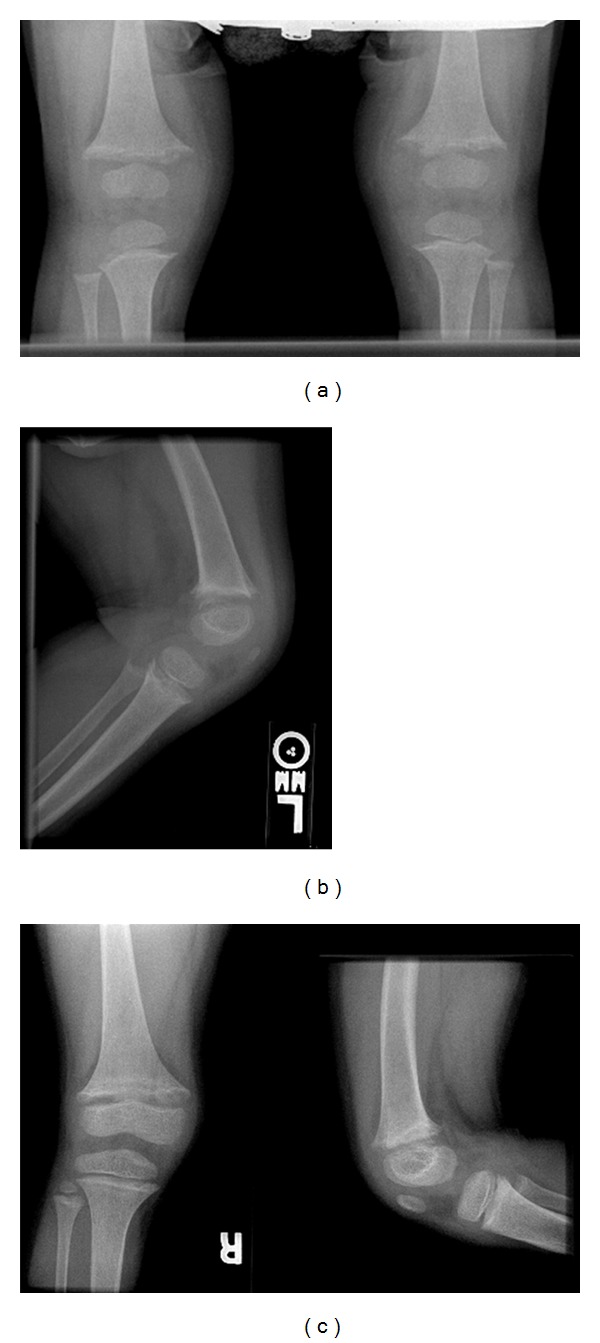
Radiographs of knees at diagnosis (a) and before phosphate and calcitriol were commenced. (a) Radiograph of both knees at 1 year of age showed irregularity, fraying and flaring of the distal femoral and proximal tibia metaphyses bilaterally. (b) Radiograph of knees at 3 years of age showed worsening rickets with increased widening, cupping and fraying of metaphyses of the distal femur, the proximal tibia, and fibula. (c) Radiograph of knees at 4 years of age showed persistent widening of metaphyses in both knees.

**Figure 2:**
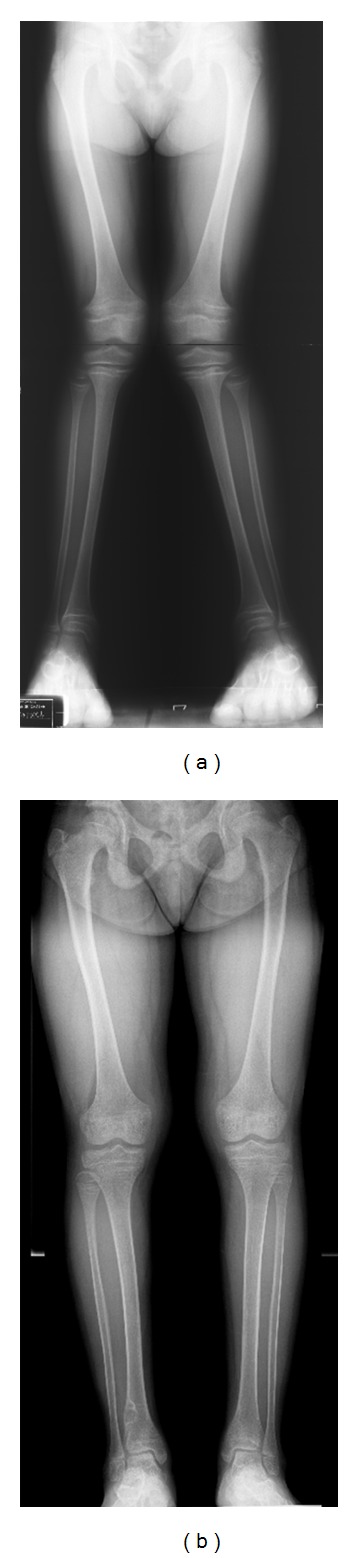
Radiograph of both lower extremities in a standing view. (a) Marked genu valgum deformity (24-degree angulation on the left and 20 degrees on the right) was noted at 5 years of age before phosphate and calcitriol therapy was commenced. (b) Marked improvement in genu valgum and healing of rickets at 10 years of age (4-degree angulation on the left and 5 degrees on the right).

A female patient, second child of nonrelated Dominican parents was born at 39 weeks' gestation after a normal uncomplicated pregnancy and a normal spontaneous vaginal delivery. Her birth weight was 4.1 kg. She developed genu varum around age 1 year. Biochemical studies showed normal serum calcium, low serum phosphorus, significantly elevated alkaline phosphatase (ALP), and parathyroid hormone (PTH) levels (Table 1). Radiographic studies of knee and wrist joints showed evidence of rickets (Figure 1). Initial diagnosis made prior to her referral to a metabolic bone specialist was vitamin D deficiency rickets. She was treated with ergocalciferol at various doses for 2 years with no improvement. Her serum 25 hydroxy vitamin D increased from 22 ng/mL at baseline to 80 ng/mL after a megadose of ergocalciferol. Repeat radiographs of her knees at age 3 years showed increased widening of physes with increased cupping and fraying of the metaphyses (Figure 2(a)). She had complaints of polyuria, polydipsia, enuresis, and bone pain.

Further laboratory investigations at age 3 years revealed mild metabolic acidosis with serum bicarbonate ranging from 19-20 mmol/L, glycosuria (glucose > 1000 mg/dL, with normal corresponding blood glucose), and proteinuria (protein > 300 mg/dL). Serum phosphorus was low at 2.2 mg/dL and serum creatinine was normal at 0.4 mg/dL, with corresponding urine phosphorus of 43.6 mg/dL and urine creatinine of 18.4 mg/dL. Tubular reabsorption of phosphorus was low at 56.9%, indicating inappropriate renal wasting of phosphate. All these findings are consistent with a proximal tubulopathy or renal Fanconi syndrome with hypophosphatemic rickets. She was started on sodium citrate and very low dose phosphate at 4 mg/kg/day at age 3 years.

She was evaluated in the genetics clinic at 5 years of age. In addition to the tubulopathy, she was also found to have mild developmental delays and hypotonia. Serum amino acids showed elevated alanine and proline, while her urine amino acids showed severe generalized aminoaciduria. Urine organic acids showed a large amount of lactic acids with no ketones. Screening for carbohydrate deficient glycoprotein disorders was performed by transferrin isoelectric focusing, with normal results. These findings suggested a mitochondrial disorder, and a muscle biopsy was obtained. Histology was essentially normal with the only noted abnormality consisting of increased lipid in the myofibers, but the respiratory chain enzyme analysis showed deficiency of complex I and milder loss of complex III activity. Genetic testing included mitochondrial DNA testing for point mutations, deletions, and complex I gene sequencing, all of which were normal. Additional testing included cranial MRI, demonstrating mild white matter loss (thinning) of the corpus callosum. Further laboratory investigations to find the cause of her renal Fanconi syndrome revealed no evidence of cystinosis, as white blood cell cystine was normal. Wilson disease and lead poisoning were ruled out based on normal serum ceruloplasmin and lead levels. Her hypophosphatemic rickets was concluded to be due to secondary de Toni-Debré-Fanconi syndrome primary to the mitochondrial disorder.

The patient was referred to the pediatric metabolic bone clinic at age 5 years. She was noted to have swelling of her knees with genu valgus deformities of 24 degrees noted on her radiograph of lower extremities (Figure 2(a)) with persistent widening of the physes in both knees (Figure 1(c)). She was started on phosphate initially at 70 mg/kg/day of elemental phosphorus and calcitriol at 30 ng/kg/day and continued sodium citrate. She had significant improvement in her genu valgum with normal growth rate following along the 5th percentile. Her biochemical studies showed remarkable improvement with normalized serum phosphorus and PTH levels and significantly decreased ALP level (Table 1). Phosphate dose was decreased to 50 mg/kg/day at age 6 years. By age 10 years, her genu valgus deformities were 4-5 degrees with healing of rickets (Figure 2(b)). She had no fractures and her bone pain resolved. Renal ultrasound showed no nephrocalcinosis. Her bone mineral density (BMD) at age 10 years showed normal lumbar BMD at 0.654 gm/cm^2^ or *Z* score at 0, while total body BMD *Z* score was low at −2.2.

## 3. Discussion

This case presents a diagnostic challenge in making a correct diagnosis that requires a thorough laboratory investigation. There are multiple causes of rickets that clinicians should consider in the differential diagnoses other than vitamin D deficiency or nutritional rickets—the most common cause, when evaluating a toddler with clinical, biochemical, and radiological rickets. Vitamin D deficiency and hypophosphatemic rickets can share similar biochemical findings of low phosphorus, elevated alkaline phosphatase, and PTH levels. Renal phosphate wasting is the main biochemical difference between these 2 causes of rickets; therefore, biochemical study to assess TRP should be obtained. In our patient, this was not done until the patient was 3 years old when her rickets was refractory to ergocalciferol therapy. Moreover, her polyuria and polydipsia should be the clues to investigate for a possible renal tubular disorder associated with metabolic bone disease. Diagnosis of renal Fanconi syndrome presenting with hypophosphatemic rickets was made on the basis of phosphaturia, glycosuria, generalized aminoaciduria, and metabolic acidosis. Other genetic causes of hypophosphatemic disorders such as XLH were unlikely with the presence of her generalized tubulopathy.

Fanconi syndrome is a generalized dysfunction of the proximal renal tubule, leading to impaired reabsorption of amino acids, glucose, phosphate, potassium, and bicarbonate with increased excretion of these solutes into the urine [2]. The classic clinical features of Fanconi syndrome include polyuria, dehydration, hypokalemia, hypophosphatemia, and metabolic acidosis. The chronic loss of phosphate and the inadequate synthesis of 1,25-OHD together result in phosphate depletion and demineralization of the bone, causing hypophosphatemic rickets in children or osteomalacia in adults [3]. Fanconi syndrome can either be inherited or acquired. The inherited form is seen in a number of genetic disorders such as cystinosis, tyrosinemia type I, glycogen storage disease type I, hereditary fructose intolerance, Lowe syndrome, Fanconi-Bickel syndrome [4], and mitochondrial disorders [5]. The acquired form is seen in association with immunologic or hematologic disorders such as multiple myeloma, Sjögren syndrome, or interstitial nephritis [6]. The acquired form has also been reported to be secondary to a number of heavy metals [7] or drugs such as aminoglycosides, valproate, ifosfamide, or antinucleoside antiretrovirals [8]. The original full name of the Fanconi syndrome is de Toni-Debré-Fanconi syndrome which was used after de Toni, Debré, and Fanconi described children with hypophosphatemic rickets and glycosuria in the 1930s [2, 9]. In some cases, the cause of Fanconi syndrome is unknown or unidentified, and thus, idiopathic Fanconi syndrome is used [10, 11].

In our patient, her very young age onset suggests a genetic form of Fanconi syndrome. The etiology was found to be secondary to mitochondrial disorder, caused by a deficiency of the respiratory chain complex I. Mitochondria perform many tasks, but one of the most important is the production of energy in the form of ATP through oxidative phosphorylation. The respiratory chain consists of five protein complexes, located on the inner membrane of the mitochondria. During the oxidation process, reducing equivalents are transferred to oxygen through the enzymatic complexes of the mitochondrial respiratory chain: complexes I, III, and IV for NADH producing substrates and complexes II, III, and IV for succinate. Since mitochondria are present in all cells (except erythrocytes), a disorder of oxidative phosphorylation can give rise to a wide range of systemic diseases in any organs or tissues. The renal manifestation of mitochondrial disease is more common in children than in adults. It may be the first sign of a mitochondrial disorder, or it may appear simultaneously with neurological and neuromuscular symptoms. Due to the high metabolic demand of cells in the proximal tubule, renal tubulopathy, or de Toni-Debré-Fanconi syndrome, is a common manifestation, predominantly as a result of a defect in the sodium-potassium-ATPase pump that drives all the transport of the solutes in the proximal tubular cells [12]. Most patients with mitochondrial disorders who manifest tubulopathy do so by the age of 2 years with earliest manifestation in some cases [13]. Multisystem diseases are almost always present in reported cases including myopathy, encephalopathy, diabetes mellitus, or cardiac involvement [14]. Mitochondrial disorders are frequently difficult to diagnose due to extreme variability in presentation, both for specific organ involvement and severity. Disease manifestation, progression, and severity are often hard to predict based on molecular and biochemical data for those presenting without a syndromic pattern [14]. At last assessment, our patient has predominantly renal involvement, with minimal manifestation of other systems.

There is no specific treatment for mitochondrial disorders, but rather a symptomatic treatment. Our patient's treatment consists of alkali supplementation in the form of sodium citrate (since she did not have hypokalemia) for her metabolic acidosis and phosphate and calcitriol for her hypophosphatemic rickets [15, 16]. This resulted in normalized serum phosphorus level without secondary hyperparathyroidism in our patient. Early treatment, before the first year of life (mean age of 0.35 years) and before clinical or radiographic evidence of rickets with phosphate and calcitriol, has been shown to improve growth and bone outcome in XLH [17]. Conversely, late treatment (mean age 2.1 years) does not completely normalize skeletal development [17]. Despite adequate medical treatment, the orthopedic outcome in XLH may be unsatisfactory and in most cases, the deformities do not resolve and require surgical correction [18]. Our patient had an excellent biochemical and radiographic response to therapy even when begun at age 5 years with improvement in her genu valgus deformity. This remarkable response suggests that the orthopedic abnormalities seen with the intrinsic renal proximal tubular defect as in Fanconi syndrome are relatively more responsive to medical treatment.

BMD findings in our patient, similar to that of XLH, showed a discrepancy between the BMD of the spine and the peripheral skeleton. The BMD is typically elevated at the lumbar spine but low at the peripheral skeleton in both treated and untreated patients with XLH [19, 20]. XLH has differential disease effect on the axial versus the appendicular skeleton, in that there is increased trabecular bone volume (prevalent at the spine) as part of the disease process, but decreased cortical bone (predominant at the peripheral skeleton), reflecting the underlying mineralization defect that is not entirely corrected by current treatment approach [21, 22].

In conclusion, we report a 10-year-old female with hypophosphatemic rickets due to de Toni-Debré-Fanconi syndrome secondary to mitochondrial respiratory chain complex I deficiency, who had excellent bone healing of rickets and improvement of her valgus deformity despite late proper medical intervention. Non-FGF23-mediated hypophosphatemic rickets may have better response to medical therapy as compared to FGF23-mediated hypophosphatemic rickets in which bone deformity continues to progress on medical therapy and surgical correction is often required.

## Figures and Tables

**Table 1:** Biochemical data over the course of followup.

Age (years)	Calcium (8–10.5 mg/dL)	Phosphorus (4.2–6.5 mg/dL)	ALP (55–380 U/L)	25-OHD (>30 ng/mL)	1,25-OHD (15–90 pg/mL)	PTH (10–65 pg/mL)	Treatment
1	10.1	3.5	1171	22	74	279	Started on ergocalciferol
2.5	8.8	2.2	1209	22	72	100	Megadose ergocalciferol
3	9.5	2.7	1153	80	126		Sodium citrate
5 (referred to bone clinic)	9.1	2.0	2049	34	35	170	Phosphate, calcitriol, and sodium citrate
6	9	4.9	886	23	64	41	Same
7	10.1	4.7	883	20	50	38	Same
8	9.1	4.7	1161	19	118	70	Same
9	8.9	2.4	1310	20	36	181	Patient nonadherent to treatment

## References

[1] Carpenter TO (2012). The expanding family of hypophosphatemic syndromes. *Journal of Bone and Mineral Metabolism*.

[2] Foreman JW, Holliday MA, Barratt TM, Avner ED (1994). Fanconi syndrome and cystinosis. *Pediatric Nephrology*.

[3] Igarashi T (2009). Fanconi syndrome. *Pediatric Nephrology*.

[4] Nyhan WL, Hoffmann GF, Zschocke J, Nyhan WL (2010). Kidney disease and electrolyte disturbances. *Inherited Metabolic Diseases : A Clinical Approach*.

[5] Munnich A, Rustin P (2001). Clinical spectrum and diagnosis of mitochondrial disorders. *The American Journal of Medical Genetics*.

[6] Rao DS, Parfitt AM, Villanueva AR, Dorman PJ, Kleerekoper M (1987). Hypophosphatemic osteomalacia and adult Fanconi syndrome due to light-chain nephropathy. Another form of oncogenous osteomalacia. *The American Journal of Medicine*.

[7] Barbier O, Jacquillet G, Tauc M, Cougnon M, Poujeol P (2005). Effect of heavy metals on, and handling by, the kidney. *Nephron Physiology*.

[8] Izzedine H, Launay-Vacher V, Isnard-Bagnis C, Deray G (2003). Drug-induced Fanconi’s syndrome. *The American Journal of Kidney Diseases*.

[9] de Toni G (1933). Remarks on the relations between renal and rickets (renal dwarfism) and renal diabetes. *Acta Paediatrics*.

[10] Patrick A, Cameron JS, Ogg CS (1981). A family with a dominant form of idiopathic Fanconi syndrome leading to renal failure in adult life. *Clinical Nephrology*.

[11] Tolaymat A, Sakarcan A, Neiberger R (1992). Idiopathic Fanconi syndrome in a family—part I: clinical aspects. *Journal of the American Society of Nephrology*.

[12] Niaudet P (1998). Mitochondrial disorders and the kidney. *Archives of Disease in Childhood*.

[13] Emma F, Giovanni M, Leonardo S, Dionisi-Vici C (2011). Renal mitochondrial cytopathies. *International Journal of Nephrology*.

[14] DiMauro S, Schon EA (2003). Mitochondrial respiratory-chain diseases. *The New England Journal of Medicine*.

[15] Rasmussen H, Pechet M, Anast C, Mazur A, Gertner J, Broadus AE (1981). Long-term treatment of familial hypophosphatemic rickets with oral phosphate and 1*α*-hydroxyvitamin D. *The Journal of Pediatrics*.

[16] Petersen DJ, Boniface AM, Schranck FW, Rupich RC, Whyte MP (1992). X-linked hypophosphatemic rickets: a study (with literature review) of linear growth response to calcitriol and phosphate therapy. *Journal of Bone and Mineral Research*.

[17] Mäkitie O, Doria A, Kooh SW, Cole WG, Daneman A, Sochett E (2003). Early treatment improves growth and biochemical and radiographic outcome in X-linked hypophosphatemic rickets. *Journal of Clinical Endocrinology and Metabolism*.

[18] Petje G, Meizer R, Radler C, Aigner N, Grill F (2008). Deformity correction in children with hereditary hypophosphatemic rickets. *Clinical Orthopaedics and Related Research*.

[19] Oliveri MB, Cassinelli H, Bergada C, Mautalen CA (1991). Bone mineral density of the spine and radius shaft in children with X-linked hypophosphatemic rickets (XLH). *Bone and Mineral*.

[20] Shore RM, Langman CB, Poznanski AK (2000). Lumbar and radial bone mineral density in children and adolescents with X-linked hypophosphatemia: evaluation with dual X-ray absorptiometry. *Skeletal Radiology*.

[21] Cheung M, Roschger P, Klaushofer K (2013). Cortical and trabecular bone density in X-linked hypophosphatemic rickets. *Journal of Clinical Endocrinology & Metabolism*.

[22] Reid IR, Murphy WA, Hardy DC, Teitelbaum SL, Bergfeld MA, Whyte MP (1991). X-linked hypophosphatemia: skeletal mass in adults assessed by histomorphometry, computed tomography, and absorptiometry. *The American Journal of Medicine*.

